# Migrant Mothers’ Acculturative Stress and Young Children’s Social Skills in South Korea: The Mediating Role of Mindful Parenting and the Moderating Roles of Multicultural Sensitivity and Social Support

**DOI:** 10.3390/bs16060940

**Published:** 2026-06-08

**Authors:** Sion Jang, Young-Eun Lee

**Affiliations:** 1 Korean Educational Development Institute (KEDI), Jincheon-gun 27873, Republic of Korea; 2 Department of Early Childhood Education, Gachon University, Seongnam-si 13120, Republic of Korea

**Keywords:** migrant mothers, acculturative stress, mindful parenting, multicultural sensitivity, social support, social skills

## Abstract

Migrant mothers experience high levels of acculturative stress while raising children in a new cultural environment. Such stress can negatively impact parenting behaviors and child development. This study draws on the Family Stress Model and ecocultural theory to examine the mediating role of mindful parenting and the moderating effects of multicultural sensitivity and social support in the relationship between acculturative stress and the social skills of young children (aged 1–7 years) of migrant mothers. Validated self-report measures were used to collect data from 338 migrant mothers residing in South Korea. The PROCESS Macro was used to analyze the data and examine the moderated mediation model. Maternal education, household income, spouse’s country of origin, and reason for migration were controlled. The results indicated that acculturative stress was associated with reduced social skills in young children due to diminished mindful parenting, which exhibited a significant mediating effect. Furthermore, multicultural sensitivity and social support each showed significant moderating effects on the respective pathways. These findings suggest that providing mindful parenting support and fostering culturally and socially supportive environments for migrant mothers can enhance the social competencies of their young children.

## 1. Introduction

### 1.1. The Rise in Immigrant Families in South Korea and the Conceptual Definition of Migrant Mothers

International migration has increased steadily worldwide over the past several decades (IOM, 2022), and South Korea is one of the countries in Asia that has experienced this shift most prominently. According to the 2024 Survey on Immigrants’ Living Conditions and Labor Force conducted by Statistics Korea, the number of foreign nationals residing in South Korea is approximately 2.65 million, accounting for 5.2% of the total population (Ministry of Justice, Republic of Korea, 2024), reflecting the rapid multicultural transformation that has unfolded over the past three decades. This shift has been substantially shaped by the international marriage migration programs led by the Ministry of Justice, Republic of Korea (2011), which facilitated the immigration of women primarily from Vietnam, China, the Philippines, and Mongolia. As a result, migrant mothers who have relocated from abroad to raise young children in South Korea, along with their families, have become an increasingly common family type in Korean society, and scholarly interest in their adaptation experiences and parenting contexts has grown accordingly.

Nevertheless, conceptual clarity regarding who constitutes a ‘migrant mother’ remains insufficient. South Korea’s Multicultural Families Support Act defines multicultural families as including foreign spouses married to Korean nationals and their children, as well as naturalized citizens and their families (K. M. Kim & Hwang, 2019). However, this legal definition excludes mothers in families formed through marriages between two foreign nationals, as well as those belonging to families of permanent residents. Consequently, a range of migrant mothers who are currently raising young children in South Korea risk exclusion from institutional support (J. Jang & Kim, 2024). In this study, therefore, a ‘migrant mother’ is operationally defined as a foreign-born woman who bears primary responsibility for raising young children in South Korea, regardless of marital status, nationality, or legal residency status. This definition encompasses diverse migration pathways, including marriage, employment, study abroad, and refugee settlement, and represents an attempt to capture the diversity of parenting contexts in immigrant families that has been insufficiently addressed in prior research.

South Korea is a society historically formed around cultural and linguistic homogeneity centered on a ‘single-ethnic nation’ identity (J. S. Kim, 2018), and migrant mothers encounter the additional psychological task of adapting to the mainstream cultural norms encapsulated in the concept of ‘Koreanness’ (Kim-Bossard, 2019). A sense of pride and identity rooted in Korean culture has influenced multicultural policies, with support for social integration tending to center on adaptation to the mainstream culture (Kudo, 2022). This context positions South Korean society as a theoretically significant research context for examining the relationship between acculturative stress and parenting processes among migrant mothers.

### 1.2. Acculturative Stress and Social Skills in Young Children

Acculturative stress refers to the psychological pressure and tension experienced during the process of adapting to a new cultural environment, encompassing negative emotions and adaptation burdens stemming from differences in language, values, and social norms (Berry, 2003). Migrant mothers in South Korea encounter diverse forms of acculturative stress, including differences in cultural norms in everyday life, language barriers, and challenges in forming social relationships (H. Oh, 2019). As migrant mothers and their children strive to establish a sense of belonging and avoid social exclusion while adapting to a new environment, they must continuously reconstruct their identities in accordance with the cultural norms of their homeland and the expectations of the host society (Berry, 2003; Sam & Berry, 2010). During this process, shifts in language and social norms can disrupt one’s sense of belonging and self-concept, leading to identity confusion and diminished self-confidence (Schwartz et al., 2010). When such psychological pressure accumulates, it may transform into physical and emotional burdens (Achotegui, 2009; Revollo et al., 2011), with reported ripple effects that extend beyond the individual psychological difficulties of the migrant mother to the emotional environment of the family system as a whole and the quality of parent–child interactions (López-Zerón et al., 2020; Masarik & Conger, 2017).

Such acculturative stress may function as a direct risk factor for the development of social skills in young children (aged 1–7 years). The social skills of young children are defined as the abilities to cooperate and regulate one’s emotions and behavior during interactions with peers and adults, and are presented as core developmental competencies that underlie subsequent school adaptation and long-term social and emotional well-being (Gresham, 2016). Research conducted with young children in South Korea found that children’s social skill levels in early childhood significantly predicted academic performance and school adjustment in the early years of elementary school (Y.-E. Lee, 2023). Among children with immigrant backgrounds, a study of preschool-aged and school-aged children from diverse immigrant backgrounds in the United States (Toro et al., 2025) reported that language barriers and cultural norm differences limited the quality of peer interactions, and a review study of children from immigrant families in the United States (Suárez-Orozco et al., 2018) consistently raised the possibility that social skill development may be constrained by social prejudice and cultural differences. However, these prior studies were primarily conducted in the immigration contexts of Western societies such as the United States, which have a comparatively long history of social exposure to multicultural diversity, and the patterns of social skill development among young children raised by migrant mothers in South Korean society, which has a relatively short history of immigrant influx and a compositionally distinct immigrant mother population, have yet to be sufficiently elucidated. Based on this theoretical rationale, the present study hypothesizes that the acculturative stress of migrant mothers will negatively influence the social skills of young children.

**Hypothesis** **1 (H1).** *Acculturative stress among migrant mothers will negatively affect the social skills of young children.*

### 1.3. Mindful Parenting as a Mediating Mechanism

The Family Stress Model (Conger et al., 2002) is a theoretical framework that explains how external socio-environmental stressors weaken parental psychological functioning, and how these changes are transmitted to children’s developmental outcomes through parenting processes. Although originally proposed in the context of economic hardship, researchers have recently called for its extension to immigrant-specific stressors such as experiences of discrimination, social exclusion, and acculturative stress (Masarik & Conger, 2017; Taylor et al., 2024). According to this model, the influence of acculturative stress on children’s development is structured not through direct pathways but through the process of depleting parental psychological resources and altering the quality and emotional tenor of parent–child interactions (Conger et al., 2002; Masarik & Conger, 2017). Indeed, studies targeting Hispanic mothers who had immigrated to the United States (Bao & Greder, 2022) and a longitudinal study targeting Hispanic parents who had immigrated to the United States (Cobb & Martínez, 2022) both reported that acculturative stress negatively affected children’s behavioral development through mediating pathways including depression and low parenting self-efficacy. In this context, mindful parenting is identified as a key mediating mechanism through which acculturative stress is transmitted to parenting behaviors.

Mindful parenting refers to a parenting orientation in which parents attend to the present moment during interactions with their child, sensitively perceive the child’s verbal and nonverbal cues, and approach both the child and their own parenting with a nonjudgmental and accepting attitude (Duncan et al., 2009). The three terms ‘mindful parenting,’ ‘mindfulness parenting,’ and ‘mindfulness-based parenting’ have been used interchangeably in the literature (Duncan et al., 2009; Kabat-Zinn, 1994); however, the present study consistently adopts the term ‘mindful parenting’ for conceptual alignment with the theoretical model and measurement instrument employed. This construct encompasses the integrated operation of multiple psychological processes, including listening with full attention, awareness of the influence of one’s own mood, nonjudgmental acceptance of parental functioning, and compassion for the child (Duncan et al., 2009). From the perspective of the Family Stress Model, mindful parenting may serve as a key mediating mechanism that attenuates the direct transmission of stress to negative parenting behaviors under conditions of cumulative stress such as acculturative stress (Lu et al., 2022; Williams et al., 2024). A study employing a mindful parenting intervention with Chinese-born parents who had immigrated to Hong Kong (Lu et al., 2022) found that mindful parenting functioned as a mechanism enabling parents to regulate negative emotional reactions and maintain positive parenting behaviors under immigration-related stress. A longitudinal study of Chinese immigrant parents who had relocated to Canada also found that acculturative stress predicted a decline in positive parenting over time (Miao et al., 2018), suggesting that mindful parenting may serve as a key mechanism for attenuating such negative transmission.

Mindful parenting is not only supportive of parents’ emotional stability but also directly linked to social skill development in young children. When parents attend to and respond acceptingly to their children’s emotional cues, young children can learn self-expression, emotion regulation, and prosocial behavior within an emotionally secure interactional context (Duncan et al., 2009). Studies conducted with mothers of young children in China (Han et al., 2023) and South Korea (E. Kim, 2023) have similarly found that mindful parenting was positively associated with children’s prosocial behavior and social skills. However, these studies addressed the relationship between mindful parenting and social skills in isolation from acculturative stress. No study to date has empirically examined mediating pathway through which acculturative stress influences the social skills of young children via mindful parenting, particularly among migrant mothers residing in South Korea. Based on these grounds, the present study establishes the following hypothesis.

**Hypothesis** **2 (H2).** *Mindful parenting will mediate the relationship between acculturative stress experienced by migrant mothers and the social skills of young children.*

### 1.4. The Moderating Roles of Multicultural Sensitivity and Social Support: An Ecocultural Theoretical Perspective

Although the Family Stress Model systematically accounts for the pathway through which acculturative stress is transmitted to children’s developmental outcomes via parenting processes, it is insufficient on its own to explain how the strength and direction of this pathway vary according to the personal and familial resources available to individuals and families. That is, an additional theoretical framework for moderating factors is needed to account for the conditions under which the negative influence of acculturative stress on mindful parenting is amplified or attenuated, as well as the resources by which the positive influence of mindful parenting on children’s social skills is augmented. Accordingly, the present study introduces ecocultural theory (Weisner, 2002) as a complementary theoretical framework.

Ecocultural theory situates children’s development within the context of the family’s daily activity settings and emphasizes that families actively construct their daily practices, values, and routines in response to the ecological and cultural resources available to them (Weisner, 2002). From this perspective, culture is understood not as a fixed individual attribute but as a broad context encompassing shared societal tasks, goals, beliefs, values, and resources. Families organize daily activities according to the type and level of available resources, and children internalize cultural values and social behavioral patterns within these lived contexts. For mothers who have migrated to South Korea, parenting takes place within a complex context in which the cultural resources of the country of origin interact with the cultural demands and supports of Korean society. Ecocultural theory provides a useful analytical framework for explaining how the personal psychological resources and social resources possessed by migrant mothers moderate the pathway through which the effects of acculturative stress are transmitted to children’s development.

Multicultural sensitivity is defined as the ability to acknowledge, accept, and respect cultural differences and to respond flexibly to intercultural encounters (Chen & Starosta, 2000), and constitutes a high-order internal competency encompassing attitudinal, affective, cognitive, and behavioral dimensions. From the perspective of ecocultural theory, multicultural sensitivity represents a core personal resource that migrant mothers can draw upon as they navigate dual cultural contexts, and plays a particularly important role in parenting processes that require the simultaneous coordination of the child’s cultural identity and the mother’s own cultural heritage (El Brashy & Miglietta, 2022). Individuals with higher multicultural sensitivity are able to regulate negative emotions arising in intercultural encounters and approach cultural differences with openness rather than defensiveness (Matsumoto et al., 2001), and this socio-emotional flexibility may operate in the direction of maintaining a stable and responsive parenting orientation even under stressful conditions. Research demonstrating that psychological resources available to parents enable adaptive parenting even under high-stress conditions (Mirosavljević & Sablić, 2025) supports the view that multicultural sensitivity, as an internal resource, may sustain mindful parenting. Furthermore, multicultural sensitivity shares the characteristic of receptive orientation toward other cultures with the integration acculturation strategy and may therefore be understood as a conceptually analogous internal resource; research showing that this strategy attenuates the negative effect of acculturative stress on parenting self-efficacy among migrant mothers residing in South Korea (Park & Bayne, 2024) supports a similar moderating effect of multicultural sensitivity. Based on these grounds, the present study establishes the following hypothesis.

**Hypothesis** **3 (H3).** *Multicultural sensitivity will moderate the relationship between acculturative stress and mindful parenting among migrant mothers, such that higher levels of multicultural sensitivity will attenuate the negative effect of acculturative stress on mindful parenting.*

If multicultural sensitivity functions as an internal psychological resource, social support constitutes the complementary core social resource. Social support is defined as the perceived availability of emotional, instrumental, and esteem-enhancing assistance from social networks including family, friends, and significant others (Zimet et al., 1988), and according to the stress-buffering model (Cohen & Wills, 1985), operates to mitigate negative effects under stressful conditions by providing emotional stability and facilitating positive cognitive reappraisal of adversity. Mothers who perceive higher levels of social support have been found to demonstrate enhanced parenting self-efficacy and more active engagement in their children’s daily lives, indirectly supporting the development of children’s social competence (Taylor et al., 2015), and were found to maintain warm parenting and respond sensitively to their children’s emotional cues even under stressful conditions (Conger et al., 2002). From the perspective of ecocultural theory, social support constitutes a social resource that provides the emotional stability and relational foundation necessary for migrant mothers to practice mindful parenting, and is expected to amplify the positive effects of mindful parenting on children’s social skills. Based on these grounds, the present study establishes the following hypothesis.

**Hypothesis** **4 (H4).** *Social support will moderate the relationship between mindful parenting and the social skills of young children among migrant mothers, such that higher levels of social support will strengthen the positive effect of mindful parenting on children’s social skills.*

### 1.5. Research Objectives and Hypotheses

**Figure 1:**
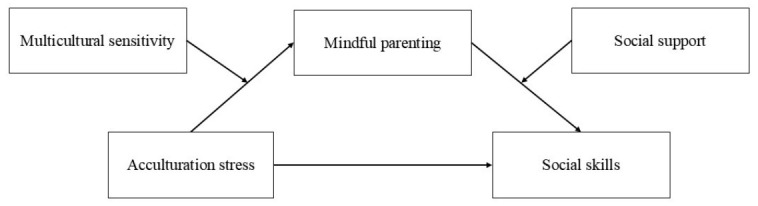
Moderated Mediation Model with Dual Moderators.

The present study examines the effects of acculturative stress among migrant mothers on the social skills of their young children, integrating the Family Stress Model (Conger et al., 2002) and ecocultural theory (Weisner, 2002) within the South Korean context. Hayes’ (2022) PROCESS Macro Model 21 is applied to simultaneously examine the mediating effect of mindful parenting and the moderating effects of multicultural sensitivity and social support (see Figure 1). Based on these theoretical frameworks, the following hypotheses were established:

**H1.**  *Acculturative stress among migrant mothers negatively affects the social skills of their young children.*

**H2.**  *Mindful parenting mediates the relationship between acculturative stress experienced by migrant mothers and the social skills of their young children.*

**H3.**  *Multicultural sensitivity moderates the relationship between acculturative stress and mindful parenting.*

**H4.**  *Social support moderates the relationship between mindful parenting and the social skills of young children.*

## 2. Method

### 2.1. Participants

The participants were 338 migrant mothers residing in South Korea. Their mean age was 34 years (standard deviation [*SD*] = 4.34) within the range of 23–50 years. In terms education, 46.2% had completed university or graduate school, 41.4% had completed high school, and 9.5% had completed middle school or less. Only 3.0% held graduate degree. Among the spouses, university graduates were the largest group (58.6%), followed by graduate degree holders (3.6%), high school graduates (33.4%), and those with the middle school education or below (4.4%). Average monthly household income was 48.1% low-income group, 19.3% in the middle-income group, and 32.6% in the high-income group. Regarding country of origin, 55.6% of the mothers were from China, and 45.6% of their spouses were from Korea.

Marriage was the primary reason for migration (71.9%). In terms of length of residence, mid-term stays were the most common (56.2%), followed by short-term (26.6%) and long-term (17.2%). Additionally 29.9% of respondents had not yet acquired Korean citizenship. Regarding employment status, full-time homemakers were the largest group (53.6%). The Seoul metropolitan area accounted for 68% of participants’ residence. For Korean language proficiency, “very proficient” was the most common response at 43.8%.

Among respondents’ children, 82.2% were only children and 17.8% had siblings. Gender distribution of children was 55.6% male and 44.4% female. The most common childcare or educational institution attended was daycare (55%). When communicating with their children, 88.2% of respondents used Korean as the primary language, more frequently than their native language. This pattern reflects the practical emphasis placed on Korean language acquisition in South Korean society, where proficiency in Korean is widely recognized as essential for children’s smooth integration into educational and social settings (W. J. Kim & Yim, 2024). The mean age of the children was 4.7 years (*SD* = 0.85, range = 1–7 years). Table 1 presents the demographic characteristics of the study participants.

### 2.2. Procedures and Instruments

As previously noted, the study participants were 338 migrant mothers residing in South Korea who were raising young children. In this study, “migrant mothers” referred to foreign-born women residing in Korea who held primary responsibility for raising their children, regardless of nationality, legal status, or migration pathway (e.g., marriage, employment, study abroad, or refugee status). Thus, the term encompassed diverse migration backgrounds and living circumstances.

Participants were recruited using convenience sampling through multicultural family support centers, local children’s centers, family support agencies, and religious communities. Additional participants were recruited through referrals from existing participants. Data were collected from 10 June to 20 August 2025. The survey was administered both online and in paper-based formats as a self-administered questionnaire to ensure accessibility and voluntary participation. Prior to participation, all respondents were informed of the study purpose, participation conditions, and the principles of voluntariness, anonymity, and confidentiality. Only participants who provided informed consent were allowed to participate. Online respondents indicated consent electronically, whereas paper-based respondents completed written consent forms before receiving the questionnaire.

A total of 347 responses were collected. Responses with more than 5% missing data or with children outside the target age range were excluded, resulting in 338 valid cases for analysis. All data were anonymized, and no personally identifiable information was collected.

Additionally, the questionnaire was developed in multiple languages to reflect the linguistic backgrounds of migrant mothers raising children in South Korea. Given that mothers from Chinese, Mongolian, and Vietnamese backgrounds represent a substantial proportion of migrant mothers residing in Korea (Statistics Korea, 2024), the questionnaire was translated into Chinese, Mongolian, and Vietnamese. An English version was also provided to enhance accessibility for participants from diverse linguistic backgrounds. To ensure conceptual and semantic equivalence across language versions, the translation process followed a systematic forward-translation and back-translation procedure. For each target language, two bilingual translators independently produced forward translations from the original Korean, and discrepancies were reviewed and resolved by consensus. Each reconciled version was then back-translated into Korean by a separate bilingual translator with no prior exposure to the original instrument, and the back-translated versions were compared with the original items to identify and correct any semantic inconsistencies. The finalized translations were subsequently reviewed by a panel of experts with backgrounds in early childhood education and multicultural family studies, who evaluated each item for clarity and cultural appropriateness, thereby supporting the content validity of the measurement items.

Content validity was assessed using a 5-point Likert scale. The Item-level Content Validity Index (I-CVI) was calculated as the proportion of experts rating each item as 4 or higher. Scale-level content validity was evaluated using both the S-CVI/Ave (mean of all I-CVI values) and the S-CVI/UA (proportion of items universally rated as 4 or higher by all experts). S-CVI/Ave values exceeded the recommended threshold of 0.78 across all five scales: multicultural sensitivity (0.86), acculturative stress (0.80), social support (0.88), mindful parenting (0.88), and social skills (0.92).

### 2.3. Measures

Acculturative stress was measured using a modified version of the instrument employed by Hong (2009), which was itself based on Sandhu and Asrabadi’s (1994) Acculturative Stress Scale for International Students (ASSIS). The subscales used in this study were culture shock (e.g., “Since moving to Korea, I feel a lot of pressure”), homesickness (e.g., “I miss my homeland and the people there”), and perceived hostility (e.g., “Korean people ridicule the values of my culture”), comprising a total of 12 items. Each item was rated on a 5-point Likert scale (1 = strongly disagree to 5 = strongly agree). Scores were computed as the mean of all 12 items, with possible scores ranging from 1 to 5, where higher scores indicating greater levels of acculturative stress. The internal reliability coefficient (Cronbach’s α) was 0.88.

Mindful parenting was measured using the Korean version of the Interpersonal Mindfulness in Parenting Scale (IM-P; Duncan, 2007), adapted by E. Kim et al. (2019) into the Korean Interpersonal Mindfulness in Parenting Scale (IM-P-K). To reflect the linguistic backgrounds of migrant mothers raising children in South Korea, questionnaires were provided in participants’ primary languages. The subscales used were listening with full attention (e.g., “When I am doing something with my child, my mind easily wanders and becomes distracted”; reverse-scored), nonjudgmental acceptance of parental functioning (e.g., “I tend to criticize myself for not being the kind of parent I want to be”), and compassion for the child (e.g., “Even when I don’t understand my child’s opinion, I try to understand their perspective”), comprising a total of 9 items. Each item was rated on a 5-point Likert scale (1 = not at all true to 5 = very true). Scores were computed as the mean of all 9 items, with possible scores ranging from 1 to 5, where higher scores indicate higher levels of mindful parenting. The internal reliability coefficient (Cronbach’s α) was 0.75.

The social skills of young children were measured using the Korean Preschool Social Skills Rating Scale (K-SSRS; Seo, 2004), validated from the Social Skills Rating System (SSRS) developed by Gresham and Elliott (1990). This study employed 22 items organized into three subscales: assertiveness (e.g., “Asks a store clerk for necessary information and assistance”), responsibility (e.g., “Avoids situations that seem likely to cause problems”; reverse-scored), and cooperation (e.g., “Voluntarily helps family members with their tasks”). Each item was rated on a 3-point Likert scale (1 = not at all true, 2 = sometimes true, 3 = often true). Scores were computed as the mean of all 22 items, with possible scores ranging from 1 to 3, where higher scores indicate greater social skill competence in the child. The internal reliability coefficient (Cronbach’s α) for the scale used in this study was 0.88.

Multicultural sensitivity was measured using a scale adapted by O. S. Kim (2008) and subsequently revised by Hwang and Kim (2023), based on the Intercultural Sensitivity Scale (ISS) developed by Chen and Starosta (2000). This study used 21 items across four subscales: interaction engagement (e.g., “I enjoy interacting with people from different cultures”), respect for cultural differences (e.g., “I respect the values held by people from cultures completely different from my own”), interaction confidence (e.g., “I feel quite confident interacting with people from cultures completely different from my own”), and interaction enjoyment (e.g., “I do not feel discouraged when with people from cultures completely different from my own”; reverse-scored). Each item was rated on a 5-point Likert scale (1 = strongly disagree to 5 = strongly agree). Scores were computed as the mean of all 21 items, with possible scores ranging from 1 to 5, where higher scores indicate greater multicultural sensitivity. The internal reliability coefficient (Cronbach’s α) for the scale used in this study was 0.94.

Social support was measured using a scale adapted from the Multidimensional Scale of Perceived Social Support (MSPSS) developed by Zimet et al. (1988), translated by D. H. Kim (2008), revised by H. Kim (2009), and subsequently used by H. S. Lee (2011). This study used 8 items across two subscales: extended family (e.g., “My family genuinely tries to help me”) and friends in the home country (e.g., “I have friends in my home country with whom I can share my joys and sorrows”). Each item was rated on a 5-point Likert scale (1 = not at all true to 5 = very true). Scores were computed as the mean of all 8 items, with possible scores ranging from 1 to 5, where higher scores indicate higher levels of perceived social support. The internal reliability coefficient (Cronbach’s α) was 0.88.

### 2.4. Sociodemographic Controls

The following sociodemographic variables were included as controls: maternal education level, average monthly household income, spouse’s country of origin, and reason for migration. Maternal education level and average monthly household income were included as indicators of socioeconomic status, given their established associations with parenting behavior and child development (Roy et al., 2019). Spouse’s country of origin was included to account for potential cultural and linguistic differences within the parental dyad (Xiang & Colson, 2018). Reason for migration was included because pre-migration factors influence the sociocultural and psychological adjustment of immigrant families, which may subsequently affect parenting processes (Wang et al., 2023). The spouse’s country of origin and reason for migration were measured on a nominal scale, and the resulting data were converted to dummy variables for analysis. The reference categories for country of origin of spouse and reason for migration were set as “Korea” and “marriage,” respectively.

### 2.5. Data Analysis

Data analysis was performed using SPSS 25.0 and the PROCESS Macro (Hayes, 2022). Pearson correlation analysis was conducted first to examine the relationships among the key variables. the PROCESS Macro Model 4 was then applied to verify the direct and indirect effects of maternal acculturative stress on the social skills of children, diated by mindful parenting.

Model 21 was utilized to validate the core model of this study. It tested a Moderated Mediation Model with Dual Moderators, in which multicultural sensitivity and social support moderated the relationships between acculturative stress and mindful parenting, and between mindful parenting and the social skills of young children. All analyses included control variables that were selected based on theoretical and empirical grounds.

The statistical significance of the mediation and moderation effects was tested using a bootstrapping procedure with 5000 resamples to allow for the calculation of 95% confidence intervals (CIs). An effect was considered statistically significant if the CI did not include zero (Hayes, 2022; S. Jang et al., 2023).

## 3. Results

### 3.1. Results of Correlation Analysis

Descriptive statistics for the key variables of this study were calculated to examine the assumption of normality. As Table 2 shows, the absolute values of skewness and kurtosis for all variables were below 2 and 7, respectively, satisfying the normality assumption (Curran et al., 1996).

To examine multicollinearity among the key variables, Pearson correlation analysis was conducted. The correlation analysis confirmed that all correlation coefficients between variables were below 0.80, indicating no multicollinearity issues (Kline, 2005). The correlation results among key variables were as follows.

### 3.2. Results of Mediated Path Analysis

To examine the mediating effect of mindful parenting on the relationship between migrant mothers’ acculturative stress and children’s social skills, this study uses the PROCESS Macro Model 4. The analysis included the following control variables: highest maternal education level, average monthly household income level, country of origin of spouse, and reason for migration.

The analysis revealed that acculturative stress exerted a significant negative total effect on children’s social skills (*B* = −0.12, *t* = −5.47, *p* < 0.001). However, the model that included mindful parenting as a mediating variable showed a decrease in the direct effect, although it remained statistically significant (*B* = −0.06, *t* = −2.53, *p* = 0.012). Acculturative stress negatively influenced mindful parenting (*B* = −0.42, *t* = −9.73, *p* < 0.001), and mindful parenting showed a significant positive effect on children’s social skills (*B* = 0.15, *t* = 5.20, *p* < 0.001).

The bootstrapped indirect effect (5000 resamples) was statistically significant, with the 95% CI not including zero, confirming the significance of the mediating effect (*B* = −0.06, 95% CI [−0.09, −0.04]; Table 3). This indicates that the negative impact of acculturative stress on the social skills of young children is partially mediated by mindful parenting.

### 3.3. Results of the Moderated Mediation Model

To examine whether multicultural sensitivity and social support moderated specific segments of the mediation path, this study used PROCESS Macro Model 21. Highest education level of the migrant mother, average monthly household income level, country of origin of spouse, and reason for migration were included as control variables.

The results showed significant positive effect of average monthly household income (*B* = 0.05, *p* < 0.01) and having a spouse from China (*B* = 0.16, *p* < 0.001) on the social skills of young children.

**Figure 2:**
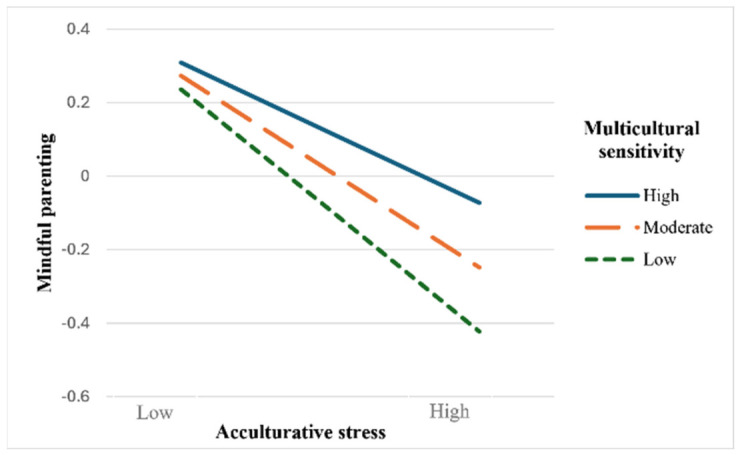
Interaction Effect of Acculturative Stress and Multicultural Sensitivity on Mindful Parenting.

Moderation analysis revealed that multicultural sensitivity significantly moderated the relationship between acculturative stress and mindful parenting (*B* = 0.12, standard error [*SE*] = 0.04, *p* = 0.007). Specifically, when multicultural sensitivity was low, maternal mindful parenting levels tended to decrease sharply as acculturative stress increased (*B* = −0.43, *SE* = 0.06, *p* < 0.001), and the negative effect persisted even at moderate levels (*B* = −0.34, *SE* = 0.05, *p* < 0.001). Conversely, when multicultural sensitivity was high, negative effects were mitigated (*B* = −0.25, *SE* = 0.06, *p* < 0.001), showing a clear weakening of the impact of acculturative stress (see Figure 2). This suggests that multicultural sensitivity is a protective factor that helps with the maintenance of mindful parenting in situations of cultural adaptation stress.

**Figure 3:**
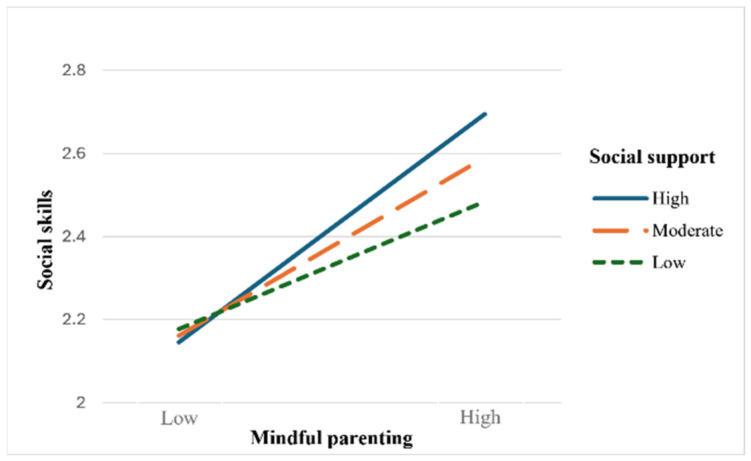
The Interaction Effect of Mindful Parenting and Social Support on the Social Skills of Young children.

Furthermore, social support significantly moderated the relationship between mindful parenting and child social skills (*B* = 0.13, *SE* = 0.05, *p* = 0.009). When social support was low, the relationship between the two variables was not significant (*B* = −0.02, *SE* = 0.06, *p* = 0.68). However, when social support was at a moderate level, a significant positive relationship emerged (*B* = 0.06, *SE* = 0.03, *p* = 0.046). When social support was high, the positive effect of mindful parenting on children’s social skills was further enhanced (*B* = 0.15, *SE* = 0.04, *p* < 0.001) (see Figure 3). This indicates that higher social support levels amplify the positive impact of maternal mindful parenting on child social skill development. The direct effect of acculturative stress on children’s social skills was found to be marginally statistically significant (*B* = −0.07, *SE* = 0.04, *p* = 0.059). This suggests that the indirect path mediated by mindful parenting partially explains the influence of acculturative stress.

Conditional indirect effects analysis revealed that the indirect effect of acculturative stress on children’s social skills was significant only at high levels of both multicultural sensitivity and social support (95% CI [0.006, 0.035]). The index of moderated mediation was also statistically significant (0.015, 95% CI [0.003, 0.034]; Table 4). This indicates that multicultural sensitivity is an internal protective factor for migrant mothers, whereas social support is an external protective factor. Each factor moderates the strength of the indirect path through which acculturative stress affects children’s social skills via mindful parenting.

## 4. Discussion

This study employed a moderated mediation model to examine the relationships among acculturative stress, mindful parenting, multicultural sensitivity, social support, and social skills in young children among migrant mothers residing in South Korea. The results supported all four hypotheses, providing important theoretical and practical implications for the parenting processes of migrant mothers and the social development of their young children.

### 4.1. Acculturative Stress and Social Skills in Young Children: Implications Within the South Korean Context

First, the acculturative stress of migrant mothers was found to exert a significant and negative effect on children’s social skills. This result supports the core premise of the Family Stress Model (Conger et al., 2002), which posits that external socio-environmental stressors deplete parental psychological resources and are transmitted to children’s developmental outcomes through parenting processes. A study targeting migrant mothers residing in South Korea (Y. Lim, 2023) found that maternal acculturative stress impeded the school adjustment of children in middle school, ultimately elevating their depressive symptom levels. A study targeting Chinese American parents in the United States (Hou et al., 2016) similarly reported that parental acculturative stress negatively affected adolescents’ depressive symptoms and delinquent behaviors by weakening the emotional bond between parent and child. This consistent pattern, observed across different immigration contexts and child age groups, whereby acculturative stress is negatively transmitted to children’s development through the depletion of parental psychological and relational resources, reinforces the significance of the present study’s finding that migrant mothers’ acculturative stress reduces young children’s social skills. The present study’s results are consistent with the body of prior literature while extending its scope to the developmental domain of social skills in young children.

These results carry three distinct implications. First, the finding that acculturative stress functions as a direct risk factor for children’s social skills demonstrates that the stress experienced by migrant mothers does not remain confined to individual psychological difficulty, but radiates through the family’s emotional environment to affect children’s developmental outcomes. Migrant mothers are continuously exposed to cultural differences in everyday life, including dining etiquette, communicative styles, and interpersonal norms (H. Oh, 2019), and the resulting accumulated stress may be transmitted to children through nonverbal emotional cues such as facial expressions, vocal tone, and physical tension, limiting the quality of experiences through which young children explore others’ emotions and participate in social interactions (Morris et al., 2007). Second, this result suggests that children’s social skills are developmental products shaped more strongly by the quality of the home environment than by individual characteristics. Social skills in early childhood are formed through the repeated accumulation of parental emotional responsiveness and interactional styles (Duncan et al., 2009), and when maternal acculturative stress weakens this responsiveness, the development of the social skill dimensions measured in this study, namely assertiveness, cooperation, and responsibility, may be constrained. Third, the finding that the effects of acculturative stress experienced by migrant mothers extend to their young children demonstrates that developmental risks for the children of immigrant families may begin to form prior to school entry. This suggests that interventions limited to the school-age period may be insufficient in supporting immigrant families, and provides grounds for the necessity of early support systems that include the preschool years.

### 4.2. The Mediating Role of Mindful Parenting

Second, mindful parenting was found to partially mediate the relationship between acculturative stress among migrant mothers and the social skills of their young children. This result is supported by a growing body of empirical evidence demonstrating that parental mindfulness functions as a key transmission mechanism through which stress affects children’s developmental outcomes. Cheung and Wang (2022) examined 172 Chinese mothers of young children residing in mainland China and Hong Kong and found that mindful parenting fully mediated the relationship between mothers’ perceived stress during the COVID-19 pandemic and children’s prosocial behavior. In their discussion, the authors interpreted these findings as indicating that stress does not impair children’s development through direct exposure alone; rather, it first degrades the quality of mothers’ present-centered attention and nonjudgmental responsiveness in everyday parent–child interactions, and it is through this deterioration in interactional quality that children’s social and behavioral functioning is subsequently affected. Taraban et al. (2025), in a longitudinal study of low-income Black and Latina mothers in the United States, further confirmed that maternal mindful parenting predicted children’s social competence and reduced internalizing and externalizing symptoms. These authors argued that the capacity to maintain an attentive, nonjudgmental, and compassionate orientation in interactions with one’s child constitutes a psychological protective resource that operates upstream of the stress-to-parenting transmission pathway, and is particularly consequential for mothers exposed to multiple chronic stressors such as economic disadvantage and minority status. Although these prior studies addressed stress arising from specific contexts such as transient social disruption or economic hardship, they share the core pathway through which stress is transmitted to children’s developmental outcomes via mindful parenting. In this respect, they support the present study’s finding that, for migrant mothers experiencing the persistent psychological pressure of acculturative stress in South Korean society, the extent to which this stress depletes mindful parenting capacity constitutes the primary pathway through which its negative effects reach children’s social skill development.

These results carry three distinct implications, interpreted through the lens of the three subscales of mindful parenting employed in the present study, namely listening with full attention, nonjudgmental acceptance of parental functioning, and compassion for the child. First, insofar as listening with full attention supports the development of assertiveness in young children, the present findings demonstrate that migrant mothers’ ability to maintain attentive awareness of their children’s verbal and nonverbal cues even under acculturative stress directly contributes to children’s social skill development. Duncan et al. (2009) theorized that listening with full attention fosters a relational environment in which children’s communicative bids are genuinely received, providing the foundation for children to initiate social exchanges and express their needs with confidence in interactions with peers and adults, and the present findings suggest that the depletion of this capacity under acculturative stress may be among the first mechanisms through which children’s developing assertiveness becomes constrained. Second, nonjudgmental acceptance of parental functioning carries important implications in that it reduces the likelihood of harsh or reactive responses to children’s behavior and provides the calm, regulated interactional environment from which children internalize norms of cooperation; Zimmer-Gembeck et al. (2022) demonstrated that parental emotion regulation constitutes a foundational mechanism linking parenting quality to children’s social outcomes, and when this nonjudgmental acceptance capacity is impaired by the persistent self-critical internal dialog that migrant mothers may experience in response to culturally incongruent parenting expectations in South Korean institutional settings, the cooperative behavioral scaffolding children develop within the home environment may be weakened (Morris et al., 2007). Third, compassion for the child functions as a mechanism through which mothers’ empathic responsiveness to children’s emotional experiences directly promotes prosocial behavior and responsibility; Zhang et al. (2026) found that parental mindfulness characterized by acceptance, kindness, and compassion was significantly associated with young children’s prosocial behavior toward peers, explaining that children internalize compassionate interactional patterns through observation and modeling, and these findings suggest that sustaining compassionate attunement even under elevated acculturative stress constitutes a direct pathway through which migrant mothers can support children’s development of cooperation and responsibility.

### 4.3. Multicultural Sensitivity: Its Role as an Internal Regulatory Resource

Third, multicultural sensitivity was found to significantly moderate the relationship between acculturative stress and mindful parenting among migrant mothers. Specifically, when multicultural sensitivity was low, the negative effect of acculturative stress on mindful parenting was amplified, whereas when it was high, this negative effect was markedly attenuated. This suggests that migrant mothers with higher multicultural sensitivity were better able to perceive the psychological pressure arising from cultural differences as a manageable challenge rather than a threat, and to maintain the capacity to regulate their emotional responses and focus on interactions with their children even under stress. These results are supported by prior research on the protective effects of personal psychological resources. The finding that the openness factor of multicultural sensitivity performs a buffering function between intercultural conflict and emotional difficulties among international high school students in China (He et al., 2023), and the finding that higher multicultural sensitivity among immigrant parents residing in Chile leads to more stable parenting participation by enabling them to convert cultural differences into parenting resources rather than perceiving them as barriers (Bilbao et al., 2025), support the moderating effect found in the present study. In particular, a comprehensive review of the literature demonstrated that mothers’ psychological resources block the negative transmission of stress to parenting behaviors (Mirosavljević & Sablić, 2025), providing empirical support for the contention that migrant mothers residing in South Korea who possess high multicultural sensitivity are able to maintain mindful parenting stably even under acculturative stress. Whereas the existing literature has primarily attended to the direct relationship between multicultural sensitivity and adaptation, the present study is distinguished by directly elucidating the moderating pathway through which multicultural sensitivity attenuates the negative effect of acculturative stress on mindful parenting. These results carry two implications. First, migrant mothers with high multicultural sensitivity were able to prevent emotional depletion during the acculturation process by perceiving cultural differences as explorable challenges, and thereby to maintain mindful parenting stably even under acculturative stress. Conversely, when multicultural sensitivity is low, this cognitive buffering fails to operate, and acculturative stress may transmit directly to parenting behaviors with amplified negative effects. Second, the integrated moderating effects of the four subscales, namely interaction engagement, respect for cultural differences, interaction confidence, and interaction enjoyment, demonstrate that multicultural sensitivity functions as a multidimensional psychological competency that protects parenting even under stress, going beyond a simple cultural attitude, which constitutes the scholarly significance of the present study.

### 4.4. Social Support: Its Role as an External Regulatory Resource

Fourth, social support was found to significantly moderate the relationship between mindful parenting and the social skills of young children. Specifically, when social support was low, the relationship between mindful parenting and children’s social skills was not significant; at moderate levels or above, a significant positive relationship emerged, and the positive effect was further amplified as support levels increased. This indicates that the more mothers perceived sufficient support from extended family and home-country friend networks, the more strongly the positive effects of mindful parenting on children’s social skill development were reinforced, and demonstrates—from the perspective of ecocultural theory (Weisner, 2002)—that social support constitutes a core social resource available to migrant mothers in their parenting processes.

These results are supported by prior research. Leahy-Warren (2005) reported that emotional and instrumental support functioned as a key pathway for strengthening parenting confidence among first-time mothers, explaining that the experience of receiving support secured mothers’ psychological capacity and formed an inner sense of stability that allowed them to focus on interactions with their children. A study targeting parents of children attending preschools and childcare centers in Japan (Hosokawa & Katsura, 2024) similarly found that higher perceived social support among parents was associated with increased prosocial behavior and decreased internalizing and externalizing problems among children, explaining that whereas insufficient social support undermines parents’ psychological health and leads to suboptimal parenting, sufficient support provides a stable foundation enabling parents to respond acceptingly to their children’s emotional cues. Particularly noteworthy in the present study is that social support was operationalized by distinguishing between two sources, namely extended family and home-country friend networks, with home-country friends constituting the primary focus of theoretical interest. For migrant mothers, home-country friend networks function as a qualitatively distinct source of support: they provide emotional reassurance rooted in shared migration experiences and cultural background, as well as instrumental assistance in navigating the gap between home-country parenting norms and the expectations of South Korean society. Research on migrant mothers with young children has documented that the loss of familiar social and cultural networks upon migration, particularly the severing of ties with co-ethnic peers and home-country friends, constitutes a primary driver of social isolation and parenting-related anxiety, and that maintaining these transnational connections functions as a protective resource for maternal psychological well-being (M. Lim et al., 2022; Yang et al., 2022). When the emotional support derived from home-country friend networks operates alongside the instrumental burden-alleviating function of extended family support, migrant mothers are interpreted as having been able to simultaneously secure the psychological capacity and relational stability necessary for mindful parenting. Whereas prior studies have primarily addressed the direct relationship between social support and parenting behaviors, the present study extends this literature by empirically demonstrating that home-country friend networks and extended family support jointly function as a moderating resource that amplifies the effects of mindful parenting on children’s social skill development in the specific context of migrant mothers in South Korea.

These results carry two implications. First, the finding that the positive relationship between mindful parenting and children’s social skills was not significant when social support was low suggests that the beneficial effects of mindful parenting may be fully realized only when an adequate external support condition is in place. This demonstrates that even when migrant mothers maintain high levels of mindful parenting, the extent to which such efforts translate into improvements in children’s social skills may be limited unless sufficient social support is simultaneously available. Second, and more specifically, these findings underscore the importance of preserving and actively sustaining migrant mothers’ connections to their home-country networks rather than treating such ties as peripheral to the resettlement process. Given that the severing of home-country social networks upon migration is among the primary sources of parenting stress and social isolation for migrant mothers in South Korean society (H. Oh, 2019; Yang et al., 2022), interventions that intentionally facilitate the maintenance of transnational peer connections, through digitally mediated support platforms, co-ethnic community programs, and culturally affirming peer network groups, constitute a core priority not merely for maternal well-being, but for the social skill development of the young children they are raising.

## 5. Limitations

The present study has the following limitations. First, the cross-sectional research design makes it difficult to establish directional relationships among variables. Acculturative stress may manifest differently depending on whether mothers are in the early stages of immigration or during long-term settlement, and social skills in early childhood are also characterized by rapid change across developmental stages. Future research should adopt longitudinal designs that track developmental trajectories from infancy through the school-age years to verify more rigorously the robustness of the mediated and moderated pathways.

Second, there is a measurement limitation in that all key variables were assessed through self-report by migrant mothers. Self-report data may be subject to social desirability bias, and constructs based on self-awareness, such as mindful parenting, may differ from observed parenting behaviors. Additionally, mothers’ reports of their young children’s social skills may differ from teacher assessments or behavioral observations. Future research should supplement the validity of measurement by employing multi-informant assessment methods such as teacher ratings and parent–child interaction observations.

Third, the study sample predominantly comprised mothers of Chinese, Vietnamese, Mongolian, and Filipino origin, reflecting the reality that these nationalities account for the majority of marriage migrant women in South Korea according to Statistics Korea’s 2024 Survey on Immigrant Residents. Moreover, the sample consisted largely of mothers who settled in South Korea primarily through marriage migration, and the parenting contexts of migrant mothers who reside in South Korea through other pathways, including employment, study abroad, and refugee settlement, were not adequately represented. Furthermore, as the present study’s findings were derived within the specific sociocultural context of South Korea, there are limitations in generalizing to other countries or immigrant groups with different immigration policies and multicultural acceptance structures. Future research should construct samples encompassing a wider range of countries of origin, migration pathways, and receiving country contexts.

Fourth, the use of convenience and snowball sampling, together with the geographic concentration of respondents in the Seoul metropolitan area (68%), limits generalizability to migrant mothers residing in rural and smaller urban areas. Future research is recommended to employ probability sampling to strengthen external validity.

## 6. Implications

The present study empirically examined the pathway through which acculturative stress among migrant mothers affects the social skills of their young children via mindful parenting, and confirmed that multicultural sensitivity and social support each function as internal and external regulatory resources, respectively. These findings are of scholarly significance in that they extend the cross-cultural applicability of the Family Stress Model (Conger et al., 2002) in the context of immigrant families and demonstrate the theoretical utility of integrating ecocultural theory (Weisner, 2002) for explaining parenting processes in immigrant households.

On the practical side, the present study’s findings provide concrete implications for the direction of parenting education and counseling programs supporting immigrant families in South Korea. Given that mindful parenting was identified as a key mediating mechanism that attenuates the negative effects of acculturative stress on parenting behaviors and directly supports children’s social skill development, integrating mindful parenting strategies into parenting education and counseling for migrant mothers may prove effective. Reports indicating that mindfulness-based parenting programs piloted at South Korea’s Multicultural Family Support Centers have received high satisfaction ratings from migrant mothers experiencing parenting burnout (Y. Kim, 2026; S. Oh, 2025) support the practical efficacy of such interventions. However, current multicultural family support programs in South Korea remain concentrated on language acquisition and cultural assimilation, and their limitations in sufficiently addressing the complex parenting needs of migrant mothers have been noted (Kudo, 2022), pointing to the need for modular programs that integratively support emotional well-being and parenting competence.

Educational interventions that enhance multicultural sensitivity can contribute to strengthening the internal resources that enable migrant mothers to maintain mindful parenting even under acculturative stress, and cultural exchange activities combined with intercultural competency programs may serve as an effective approach for cultivating migrant mothers’ abilities to flexibly navigate the cultural boundaries between their countries of origin and South Korea. In addition, programs that assist in forming and sustaining informal social support networks with spouses, relatives, and fellow parents carry practical implications in that they strengthen the social resources that amplify the effects of mindful parenting.

At the policy level in South Korea, the present study emphasizes the need for a multilayered support system encompassing individual, family, home-country friend network, community, and national dimensions. An integrated approach that combines mindfulness-based psychological support, multicultural sensitivity education, and social network formation support with institutional and financial foundations can contribute substantively to improving the parenting environment of migrant mothers, where language barriers, cultural isolation, parenting conflicts, and economic instability operate in complex interaction. Such a support system lays the groundwork for children with immigrant backgrounds to grow up healthily and integrate as members of South Korean society, which is in alignment with the international discourse on child and adolescent welfare.

## 7. Conclusions

Integrating the Family Stress Model and ecocultural theory, the present study was conducted within the unique context of South Korea, a society with a comparatively short history of immigrant influx and a predominance of marriage migration. Through this integration, the study empirically examined the moderated mediation pathway through which migrant mothers’ acculturative stress influences young children’s social skills via mindful parenting, with multicultural sensitivity and social support functioning as internal and external protective resources, respectively. These findings contribute to expanding understanding of parenting processes and early childhood development in immigrant families within the East Asian immigration context, which has been insufficiently addressed in Western and Chinese immigration research. Furthermore, the results are expected to provide important implications for the development of policies and intervention programs aimed at supporting the well-being of migrant mothers and their children in rapidly diversifying societies.

## Figures and Tables

**Table 1:** Demographic Characteristics of Participants (*n* = 338).

Variable	% (*n*)	Variable	% (*n*)
Parental characteristics			
Migrant mother educational attainment		Spouse country of origin	
Middle school or below	9.5 (32)	Korea	45.6 (154)
High school graduate	41.4 (140)	Vietnam	6.5 (22)
College or university degree	46.2 (156)	China	36.1 (122)
Graduate or postgraduate degree	3 (10)	Mongolia	4.4 (15)
Spouse’s educational attainment		Philippines	1.2 (4)
Middle school or below	4.4 (15)	Other	6.2 (21)
High school graduate	33.4 (113)	Duration of residence in South Korea	
College or university degree	58.6 (198)	Recent migrants (<5 years)	26.6 (90)
Graduate or postgraduate degree	3.6 (12)	Mid-term residents (5–9 years)	56.2 (190)
Reason for migration to South Korea		Long-term residents (≥10 years)	17.2 (58)
Marriage	71.9 (243)	Employment status	
Study	6.5 (22)	Full-time homemaker	53.6 (181)
Immigration (other)	2.7 (9)	Part-time	17.5 (59)
Employment	18.9 (64)	Full-time employment	29 (98)
Acquisition of South Korean citizenship		Korean language proficiency	
Acquired	70.1 (237)	Very proficient	43.8 (148)
Not acquired	29.9 (101)	Moderate	49.4 (167)
Country of origin		Limited	2.7 (9)
Vietnam	34 (115)	Not proficient at all	4.1 (14)
China	55.6 (188)		
Mongolia	4.7 (16)		
Philippines	3.8 (13)		
Other	1.8 (6)		
Monthly household income			
Less than ₩1,000,000 (≈US $740)	0.3 (1)		
₩1,010,000–₩2,000,000 (≈US $750–$1480)	2.4 (8)		
₩2,010,000–₩3,000,000 (≈US $1490–$2220)	27.2 (92)		
₩3,010,000–₩4,000,000 (≈US $2230–$2960)	18 (61)		
₩4,010,000–₩5,000,000 (≈US $2970–$3700)	19.2 (65)		
Over ₩5,000,000 (≈US $3700 or above)	32.8 (111)		
Child characteristics			
Presence of siblings		Primary language used with child	
Only child	82.2 (278)	Korean	88.2 (298)
With siblings (including twins)	17.8 (60)	Vietnamese	3 (10)
Sex		Chinese	5 (17)
Male	55.6 (188)	Mongolian	3.3 (11)
Female	44.4 (150)	Other	0.6 (2)
Type of institution attended			
Childcare center	55 (186)		
Kindergarten	42 (142)		
Elementary school	3 (10)		

**Table 2:** Descriptive Statistics and Correlations Between Primary Variables.

Variable	*M*	*SD*	1	2	3	4	5
1. Acculturative stress among migrant mothers	2.17	0.76	1				
2. Mindful parenting	3.78	0.69	−0.41 **	1			
3. Social skills of young children	2.39	0.35	−0.26 **	0.29 **	1		
4. Multicultural sensitivity	3.9	0.76	−0.18 **	0.28 **	0.76 **	1	
5. Social support	4.06	0.7	0.6	0.17 **	0.59 **	0.74 **	1

*M*, mean; *SD*, standard deviation. ** *p* < 0.01.

**Table 3:** Results of Moderated Mediation Model (*n* = 338).

Predictor	*B*	SE	*t*	*p*	*LLCI*	*ULCI*
Outcome: Mindful parenting						
Constant	4.49	0.23	19.43	<0.001	4.04	4.95
Acculturative stress	−0.42	0.04	−9.73	<0.001	−0.5	−0.33
Migrant mother education	0.19	0.05	4.06	<0.001	0.1	0.28
Monthly household income level	−0.08	0.03	−3.15	0.002	−0.13	−0.03
(Spouse country and reason for migration: ns)	—	—	—	—	—	—
Outcome: Child social skills						
Constant	1.82	0.17	10.45	<0.001	1.48	2.17
Acculturative stress	−0.06	0.02	−2.53	0.012	−0.11	−0.01
Mindful parenting	0.15	0.03	5.2	<0.001	0.09	0.2
Migrant mother education	−0.12	0.02	−4.81	<0.001	−0.17	−0.07
Monthly household income level	0.13	0.01	9.55	<0.001	0.10	0.15
(Spouse country and reason for migration: ns)	—	—	—	—	—	—
Indirect effect	−0.06	0.01	—	—	−0.09	−0.04

Control variables included migrant mother’s education, average monthly household income, country of origin of spouse (dummy-coded, reference = Korea), and reason for migration (dummy-coded, reference = marriage). Bootstrap sample = 5000. Ninety-five percent confidence intervals (CIs) are percentile bootstrap CIs.

**Table 4:** Results of Moderated Mediation Model with Dual Moderators (*n* = 338).

	*B*	*SE*	*t*	*p*	*LLCI*	*ULCI*
Regression for mindful parenting						
Constant	−0.14	0.21	−0.66	0.51	−0.55	0.27
Acculturative stress	−0.34	0.05	−6.96	<0.001	−0.44	−0.25
Multicultural sensitivity	0.14	0.06	2.17	0.031	0.01	0.26
Acculturative stress × Multicultural sensitivity	0.12	0.04	2.73	0.007	0.03	0.21
Migrant mother education	0.2	0.06	3.3	0.001	0.08	0.32
Monthly household income level	−0.1	0.03	−3.02	0.003	−0.17	−0.04
(Spouse country and migration reason: ns)	—	—	—	—	—	—
Regression in social skills						
Constant	2.21	0.10	21.33	<0.001	2	2.41
Acculturative stress	−0.07	0.04	−1.90	0.059	−0.14	0.00
Mindful parenting	0.07	0.03	2.01	0.046	0.00	0.13
Social support	0.31	0.04	8.29	<0.001	0.23	0.38
Mindful parenting × Social support	0.13	0.05	2.61	0.009	0.03	0.22
Migrant mother education	−0.02	0.03	−0.077	0.44	−0.08	0.03
Monthly household income	0.05	0.01	3.43	0.001	0.02	0.08
(Spouse country and migration reason: ns)	—	—	—	—	—	—
Conditional indirect effects of acculturative stress on child social skills						
Low multicultural sensitivity (−1 SD)	−0.43	0.06	—	—	−0.54	−0.32
Mean multicultural sensitivity	−0.34	0.05	—	—	−0.44	−0.25
High multicultural sensitivity (+1 SD)	−0.25	0.06	—	—	−0.37	−0.13
Low social support (−1 SD)	−0.02	0.06	—	—	−0.13	0.09
Mean social support	0.07	0.03	—	—	0.00	0.13
High social support (+1 SD)	0.15	0.04	—	—	0.08	0.22
Index of moderated mediation	0.02	0.01	—	—	0.00	0.03

HC3-robust standard errors were reported. All continuous variables were mean-centered. Covariates included maternal education, average monthly household income level, country of origin of spouse (dummy-coded, reference = Korea), and reason for migration (dummy-coded, reference = marriage). Bootstrap sample = 5000. 95% confidence intervals (CIs) are percentile bootstrap CIs. *LLCI*, lower limit of confidence interval; *SD*, standard deviation; *SE*, standard error; *ULCI*, upper limit of confidence interval.

## Data Availability

The data presented in this study are available upon request from the corresponding author owing to privacy concerns.
